# Serum prostate-specific antigen levels and type of work in tire manufacturing workers

**DOI:** 10.1186/s40557-014-0050-z

**Published:** 2014-11-04

**Authors:** Soo-Hyeon Kim, Keun-Ho Jang, Won-Ju Park, Do-Hyeong Kwon, Won-Yang Kang, Hyeong-Min Lim, Jai-Dong Moon

**Affiliations:** Department of Occupational and Environmental Medicine, Chonnam National University Hwasun Hospital, Hwasun, Korea; Department of Occupational and Environmental Medicine, Mokpo Christian Hospital, Mokpo, Korea

**Keywords:** Occupations, Physical activity, Prostate-specific antigen, Sedentary lifestyle

## Abstract

**Objectives:**

This study measures serum prostate-specific antigen (PSA) levels in tire-manufacturing workers, and attempts to find occupational or non-occupational factors that related to their PSA levels.

**Methods:**

A total of 1,958 healthy male workers (1,699 were production workers and 259 were office workers) took PSA measurement for analysis.

**Results:**

After adjusting for age, body mass index, hypertension, regular exercise, alcohol drinking and smoking, which were significantly related to serum PSA levels or known related factors of serum PSA levels, the geometric mean PSA levels were significantly high in the office workers (p = 0.017), the older age group (p < 0.001), the group with hypertension (p = 0.046) and the group of individuals that do not exercise regularly (p = 0.015) and the office workers were more likely to have a serum PSA level of ≥4.0 (OR 7.73, 95% CI: 2.78-21.46) or 2.5 ng/mL (OR 2.74, 95% CI: 1.49-5.08). After stratifying by age and adjusting aforementioned covariates, office workers 50 years of age and older had the significantly higher geometric mean PSA levels (p = 0.017) and were more likely to have a serum PSA level of ≥4.0 ng/mL (OR 12.90, 95% CI: 3.65-45.64) or 2.5 ng/mL (OR 3.90, 95% CI: 1.64-9.25) than production workers 50 years of age and older.

**Conclusions:**

This study showed that serum PSA levels were significantly higher among the group with hypertension or the group of individuals that did not exercise regularly or group of office workers who were considered to have lesser physical activities.

## Introduction

Prostate cancer is one of the fastest increasing cancers for the last twenty years. It is the most commonly diagnosed solid cancer among men in Europe and the United States [1-4]. Prostate cancer has the second highest mortality rate, right next to lung cancer in the United States. In the 2000s, the incidence rate has steadily elevated in Korea as well. The incidence of prostate cancer was approximately 28 out of 100,000 male people according to Korea Central Cancer Registry 2011 report in Korea. Prostate cancer is the fifth highest male cancer in Korea [5]. One of the most important tests in the diagnosis of prostate cancer is serum prostate-specific antigen (PSA) test. PSA is glycoprotein produced in the prostate gland, which increases in cases of prostate cancer. PSA with its high sensitivity is used in the screening test for prostate cancer [6,7].

Since PSA test was introduced in the United States in 1990, PSA test has been widely used for 57% of males 50 years old or older in the United States in 2001 [8]. Owing to such extensive utilization, the number of prostate cancer diagnosis elevated enormously in the past twenty years. Currently, PSA test has become an indispensable tool in the diagnosis of prostate cancer and follow-up observations after treatment [6,9]. However, in addition to prostate cancer, PSA levels may also increase in other benign diseases of the prostate gland, such as benign prostate hyperplasia or prostatitis, indicative of high false-positive rates of PSA tests. Certain investigational results indicated diagnosis of prostate cancer in only 26% of patients with an elevated PSA level [10,11]. In addition, several studies reported that PSA levels might be affected by age, physical activity, tobacco smoking, alcohol consumption and body mass index, other than diseases of the prostate gland [12-16].

The tire industry is a comprehensive, large-scale assembly industry that deals with rubber production and processes for tire manufacturing. Production employees working at these manufacturing plants are exposed to harmful chemicals, such as rubber fumes or organic solvents that can cause health impairment.

Owing to working in an environment largely at a desk, the extent of physical activities of office workers are lower than that of production workers. A previous study also revealed that the level of physical activities of office workers had been significantly lower than that of production workers [17]. Thus, this study attempts to analyze serum PSA levels focusing on tire manufacturing workers on the assumption that there will be differences in the serum PSA levels secondary to variances in occupational characteristics between office workers and production workers.

## Materials and methods

### Subjects

This study first selected a total of 1,966 male tire manufacturing workers 30 years old or older, who underwent health examinations at a general hospital from October 2013 through December 2013. This study enrolled a total of 1,958 subjects with no malignancy of prostate, excluding those 8 individuals with either omitted data. Those subjects finally-included in this study who participated the tests conducted for the purpose of health examinations and diagnosis. Each subject provided written, informed consent before participating. Physicians themselves explained the purpose, method and precautions of this study, and tests were conducted after obtaining an informed consent from each subject. Also, the study protocol was approved by the institutional review board of Chonnam National University Hwasun Hospital (IRB No. CNUHH-2014-123).

### Study variables and measurements

Subjects were interviewed by a clinician prior to the test, and each subject’s information such as age, smoking status, alcohol consumption, past history hypertension, diabetes mellitus, and occupational history were obtained through questionnaire. Height and body weight were measured on bare feet without the load of their shoes. Blood pressure was measured at a sitting position after subjects had been relaxed for ten minutes. Systolic and diastolic blood pressures were measured from the right upper arm. Their body mass index was calculated by weight (kg)/height^2^ (m^2^). The waist circumference was measured with a tape-measure from the navel around the circumference of the waist at an erect position. All subjects fasted for 12 hours or more before undergoing measurements of serum PSA, glucose, total cholesterol, triglyceride, and high-density lipoprotein cholesterol. The chemiluminescence assay was used for PSA measurements. Regarding alcohol consumption among their general characteristics, subjects with alcohol drinking of 3 cups or more a day and 2 days or more a week, regardless of alcohol variety were classified as heavy drinkers.

Subjects were classified into two groups according to their works. Subjects who were engaged in extrusion process, mixing process, building process, calendaring process, bead process, curing process, inspection process, product management and factory maintenance were classified as production workers and subjects of other occupations who worked sedentarily during most of their work time were classified as office workers. With respect to smoking status, subjects who never smoked (never-smoked) and those ex-smokers who would not smoke currently (ex-smoker) were classified as non-smokers. Subjects who would presently smoke were classified as current smokers. Group of subjects, who did exercises to the extent of sweating for 30 minutes and longer three times a week, were classified as the regular exercise group. Cases on a current antihypertensive regimen or cases with blood pressure of 140/90 mmHg or higher were defined as hypertensive. Subjects with infliction of diabetes mellitus were defined as cases that were treated for diabetes mellitus or cases with measured serum glucose levels of 126 mg/dL or higher. Also, the subjects were classified based on the high level of Adult Treatment Panel III (ATP III) classification for total cholesterol and the standard level of metabolic syndrome for waist circumference, body mass index, triglyceride and high density lipoprotein cholesterol, based on the standard of the National Cholesterol Education Program (NCEP).

### Statistical analysis

Collected data were organized and saved in a personal computer. Owing to the fact that the PSA levels did not show a normal distribution pattern, the geometric mean and geometric standard deviation were calculated after carrying out natural log transformation.

Subjects were divided into the group with production workers and another group with office workers with respect to general characteristics. A Chi-Square Test was used to make comparisons and analysis with respect to age, body mass index, waist circumference, hypertension, diabetes mellitus, level of total cholesterol, triglyceride, high-density lipoprotein cholesterol, alcohol consumption, smoking status, and regular exercise. The Student *T*-test and the Analysis of variance (ANOVA) were used to compare serum PSA levels in accordance with subject’s age, body mass index, waist circumference, hypertension, diabetes mellitus, levels of total cholesterol, triglyceride, high-density and lipoprotein cholesterol, as well as alcohol consumption, smoking status and regular exercise.

In an attempt to control the effect of covariate with respect to hypertension, (which showed significant difference in univariate analysis) and regarding age, alcohol consumption, smoking status and body mass index (which were reported to have an effect on serum PSA levels), and to make comparisons of quantitative differences in serum PSA levels for job type with age stratification and other factors, the analysis of covariance (ANCOVA) was utilized. Logistic regression was used to estimate the odds of an elevated PSA level (4.0 or 2.5 ng/mL) based on job type with age stratification and adjustment of aforementioned covariates. A two-tailed test was used for all analyses and *p* values less than 0.05 were regarded as statistically significant. All statistical tests were performed using SPSS version 18.0 (SPSS, Inc., Chicago, IL, USA).

## Results

### General characteristics of the subjects

A total of 1,958 subjects were enrolled in this investigation. The mean age of office workers was 42.9 years while that of production workers was 46.3 years, showing a significantly older age for production workers. 111 office workers (42.9%) in their 30s and 792 productions workers (46.6%) in their 40s made up the majority of each group of workers (p = 0.008). With respect to overweight, 130 office workers (50.2%) and 686 production workers (40.4%) had body mass index of 25 kg/m^2^ or greater, revealing that office workers had significantly a higher proportion (p = 0.009).

Subjects with elevated total cholesterol (≥240 mg/dl) were 25 office workers (9.7%) and 94 production workers (5.5%) and the proportion of subjects with elevated total cholesterol for office workers was significantly high (p = 0.007). 44 office workers (17.0%) and 437 production workers (25.8%) did their exercises regularly. The proportion of production workers who did exercises regularly was significantly high (p = 0.018). There were no significant differences in the proportions of subjects with elevated waist circumference, hypertension, diabetes mellitus, elevated triglyceride, elevated high density lipoprotein cholesterol, current smoking, heavy drinking according to job type (Table 1).

**Table 1:** **General characteristics of the subjects**

	**Production workers (n = 1,699)**		**Office workers (n = 259)**		**p-value** ^*^
	**n**	**(%)**	**n**	**(%)**	
Age (years)					
30-39	291	(17.1)	111	(42.9)	0.008
40-49	792	(46.6)	78	(30.1)	
50-	616	(36.3)	70	(27.0)	
Waist circumference (cm)					
<90	1,268	(74.6)	181	(69.9)	0.105
≥90	431	(25.4)	78	(30.1)	
Body mass index (kg/m^2^)					
<25	1,013	(59.6)	129	(49.8)	0.009
≥25	686	(40.4)	130	(50.2)	
Hypertension					
No	1,519	(89.4)	226	(87.3)	0.304
Yes	180	(10.6)	33	(12.7)	
Diabetes mellitus					
No	1,538	(90.5)	244	(94.2)	0.057
Yes	161	(9.5)	15	(5.8)	
Total cholesterol (mg/dL)					
<240	1,605	(94.5)	234	(90.3)	0.007
≥240	94	(5.5)	25	(9.7)	
Triglyceride (mg/dL)					
<150	995	(58.6)	151	(58.3)	0.936
≥150	704	(41.4)	108	(41.7)	
HDL-cholesterol^†^ (mg/dL)					
≥40	1,327	(78.1)	200	(77.2)	0.404
<40	372	(21.9)	59	(22.8)	
Current smoking					
No	894	(52.6)	146	(56.4)	0.253
Yes	805	(47.4)	113	(43.6)	
Heavy drinker					
No	1,262	(74.3)	183	(70.7)	0.212
Yes	437	(25.7)	76	(29.3)	
Regular exercise					
No	1,262	(74.2)	215	(83.0)	0.018
Yes	437	(25.8)	44	(17.0)	

^*^P value was calculated by chi-square test.

^†^HDL-cholesterol: High density lipoprotein cholesterol.

### Comparisons of blood prostate-specific antigen levels by variables of the subjects

Factors that related to the serum PSA levels in univariate analysis of geometric mean were age and hypertension (p = 0.001). The difference between workers with hypertension and patients without hypertension was statistically significant (p = 0.016). After adjusting for age, body mass index, hypertension, smoking, drinking alcohol and regular exercise, variables that related to serum PSA levels significantly were age (p < 0.001), hypertension (p = 0.046), regular exercise (p = 0.015) (Table 2).

**Table 2:** **Comparison of serum prostate-specific antigen levels by variables of the subjects**

	**n (%)**	**Serum PSA (ng/mL)**			
		**Geometric mean ± GSD** ^*^	**p-value** ^**†**^	**Adjusted geometric mean**	**p-value** ^**‡**^
Age (years)					
30-39	402 (20.5)	0.728 ± 1.740	0.001	0.722	<0.001
40-49	870 (44.4)	0.720 ± 1.820		0.719	
50-	686 (35.1)	0.815 ± 1.916		0.823	
Waist circumference (cm)					
<90	1,449 (74.0)	0.758 ± 1.845	0.521	0.761	0.265
≥90	509 (26.0)	0.743 ± 1.835		0.735	
Body mass index (kg/m^2^)					
<25	1,142 (58.3)	0.756 ± 1.844	0.843	0.757	0.806
≥25	816 (41.7)	0.752 ± 1.840		0.751	
Hypertension					
No	1,745 (89.1)	0.745 ± 1.830	0.016	0.747	0.046
Yes	213 (10.9)	0.829 ± 1.928		0.816	
Diabetes mellitus					
No	1,782 (91.0)	0.757 ± 1.832	0.412	0.758	0.225
Yes	176 (9.0)	0.728 ± 1.952		0.716	
Total cholesterol (mg/dL)					
<240	1,839 (93.9)	0.753 ± 1.848	0.764	0.754	0.755
≥240	119 (6.1)	0.766 ± 1.757		0.767	
Triglyceride (mg/dL)					
<150	1,146 (58.5)	0.757 ± 1.848	0.694	0.760	0.512
≥150	812 (41.5)	0.749 ± 1.757		0.747	
HDL cholesterol^§^ (mg/dL)					
≥40	1,527 (78.0)	0.760 ± 1.856	0.093	0.761	0.202
<40	431 (22.0)	0.736 ± 1.811		0.730	
Current smoking					
No	1,040 (53.1)	0.744 ± 1.877	0.314	0.743	0.235
Yes	918 (46.9)	0.765 ± 1.802		0.768	
Heavy drinker					
No	1,445 (73.8)	0.746 ± 1.847	0.194	0.746	0.157
Yes	513 (26.2)	0.777 ± 1.829		0.780	
Regular exercise					
No	1,477 (75.4)	0.763 ± 1.831	0.096	0.764	0.015
Yes	481 (24.6)	0.723 ± 1.878		0.722	

^*^GSD: geometric standard deviation.

^†^P value was calculated by *T*-test or ANOVA.

^‡^P value was calculated by ANCOVA, adjusted for age, body mass index, hypertension, current smoking, heavy drinker, regular exercise.

^§^HDL-cholesterol: High density lipoprotein cholesterol.

### Comparison of serum prostate-specific antigen levels by work type of the subjects

The geometric mean of serum PSA levels of office workers was significantly higher than that of production workers in total subjects (p = 0.014) and in subjects 50 years of age and older (p <0.001). After adjusting for each covariate, the adjusted geometric mean of office workers was significantly higher than that of production workers, which showed a significantly higher PSA level for office workers among total subjects (p = 0.017) and subjects 50 years of age and older (p <0.001) (Table 3).

**Table 3:** **Comparison of serum prostate-specific antigen levels by work type of the subjects**

	**n (%)**	**Serum PSA (ng/mL)**			
		**Geometric mean ± GSD** ^*^	**p-value** ^**†**^	**Adjusted geometric mean**	**p-value** ^**‡**^
All					
Production workers	1,699 (86.8)	0.745 ± 1.820	0.014	0.738	0.017
Office workers	259 (13.2)	0.814 ± 1.740		0.813	
Age < 50					
Production workers	1,699 (86.8)	0.722 ± 1.810	0.842	0.723	0.924
Office workers	259 (13.2)	0.729 ± 1.711		0.727	
Age ≥ 50					
Production workers	1,699 (86.8)	0.794 ± 1.888	<0.001	0.738	<0.001
Office workers	259 (13.2)	1.033 ± 2.083		0.813	

^*^GSD: geometric standard deviation.

^†^P value was calculated by *T*-test.

^‡^P value was calculated by ANCOVA, adjusted for age, body mass index, hypertension, current smoking, heavy drinker, regular exercise.

### Odds ratios of PSA elevation according to work type of the subjects

After adjusting for the covariates, there was a statistically significant trend of a higher likelihood of having serum PSA levels of ≥4.0 ng/mL with office workers (OR 7.73, 95% CI: 2.78-21.46). When the subjects were stratified by age, similar trend was observable only among the subjects 50 years of age and older (OR 12.90, 95% CI: 3.65-45.64). In addition, there were similar trends with serum levels PSA of ≥2.5 ng/mL among total subjects (OR 2.74, 95% CI: 1.49-5.08) and subjects 50 years of age and older (OR 3.90, 95% CI: 1.64-9.25) (Table 4).

**Table 4:** **The odds of having a serum PSA level of ≥4.0 or 2.5 ng/mL based on work type of the subjects**

	**PSA < threshold**	**PSA ≥ threshold**	**Crude OR**	**95% CI**	**Adjusted OR**	**95% CI** ^*^
	**n (%)**	**n (%)**				
PSA threshold 4.0 ng/mL						
All						
Production workers	1,690 (99.5)	9 (0.5)	1.00		1.00	
Office workers	251 (96.9)	8 (3.1)	5.98	2.29-15.65	7.73	2.78-21.46
Age < 50						
Production workers	1,079 (99.6)	4 (0.4)	1.00		1.00	
Office workers	187 (99.5)	1 (0.5)	1.44	0.16-12.97	2.372	0.24-22.84
Age ≥ 50						
Production workers	611 (99.2)	5 (0.8)	1.00		1.00	
Office workers	64 (90.1)	7 (9.9)	13.37	4.13-43.33	12.90	3.65-45.64
PSA threshold 2.5 ng/mL						
All						
Production workers	1,655 (97.4)	44 (2.6)	1.00		1.00	
Office workers	244 (94.2)	15 (5.8)	2.31	1.27-4.21	2.74	1.49-5.08
Age < 50						
Production workers	1,058 (97.7)	25 (2.3)	1.00		1.00	
Office workers	182 (96.8)	6 (3.2)	1.40	0.57-3.44	1.89	0.74-4.85
Age ≥ 50						
Production workers	597 (96.9)	19 (3.1)	1.00		1.00	
Office workers	62 (87.3)	9 (12.7)	4.56	1.98-10.51	3.90	1.64-9.25

^*^Odds ratio and 95% confidence intervals estimated using logistic regression model adjusted for adjusted for age, body mass index, hypertension, current smoking, heavy drinker, regular exercise.

## Discussion

The results of univariate analysis showed that the older the age group, the more likely an individual would have a higher serum PSA level. This also revealed that office workers had a significantly higher PSA level in comparison with that of production workers. The likelihood of having elevated PSA levels and the serum PSA levels of office workers were higher than those of production workers even after adjusting for age, smoking status, body mass index, hypertension, alcohol consumption, and regular exercise. Stratifying age group, similar trends were found for those in the subjects 50 years of age and older.

PSA is a glycoprotein produced by the epithelial cells in the prostate gland. It is known that the PSA level increases in prostate cancer and various benign diseases, such as benign prostate hyperplasia or prostatitis [10,11]. In addition to disorders of the prostate gland, other factors that related to serum PSA levels are age, smoking status, alcohol consumption, extent of physical activities and body mass index as suggested from previous studies [12-16]. In this study, both the current smoker group and heavy drinker group also had elevated serum PSA levels, but such increases were statistically insignificant. The group with regular physical activities was statistically shown to have significantly lower serum PSA levels, which concurred with the results of previous studies. Owing to occupational attributes, office workers work many hours sitting at a desk and have relatively less physical activities associated with their jobs. A study, which measured levels of physical activities of office workers by using a tool for objective measurement of physical activities, reported that they worked while sitting at a desk for two-thirds of their working hours (65%) [18,19].

Together, reviewing studies that compared physical activities at a job between office workers and production workers, it was suggested that physical activities of office workers were less than those of production workers [17,20]. 5α-reductase induces transformation of testosterone, major growth regulator of prostate and a precursor to Dihydrotestosterone (DHT), which is an important hormone that affects PSA production [21,22]. In times of extensive physical activities, inhibition of 5α-reductase and reduction in the level of testosterone, slow prostate growth and decrease the levels of DHT, which in turn may decrease PSA levels. Such mechanism may explain the logic behind the differences in PSA levels depending on occupational attributes demonstrated in this investigation [22,23].

According to prospective cohort studies conducted on the levels of PSA and development of prostate cancer among patients without a disorder of the prostate gland, the higher the baseline PSA levels, the more likely the patients would have a high risk of prostate cancer [24,25]. Furthermore, in a study involving only subjects with a normal serum PSA level (PSA <4.0 ng/mL), individuals with a high serum PSA level were associated with a high incidences of prostate cancer [26]. In this study, serum PSA levels were largely within normal levels. Nevertheless, in consideration of aforementioned investigation, the prospect might be that the risk of prostate cancer of office workers would be higher than that of production workers. Some previous studies reported results that concurred with the suggestion that group with low occupational physical activities would have a higher risk of developing prostate cancer as opposed to the group with high physical activities might have on the job [27-30].

The limitation of this study was, along with the limit of a cross-sectional investigation, the fact that other health factors, such as benign prostate disorders (benign prostate hyperplasia, prostatitis and others), might affect serum PSA levels, could not be completely controlled. Also, owing to the aspect that this study focused on subjects at certain workplaces of certain occupations, generalization of these results for entire groups of population may be somewhat unreasonable. Moreover, it was limitation that chemical exposures and occupational physical activity of workers which were considered to affect serum PSA levels could not be directly measured and their effect to serum PSA was assessed indirectly by analyzing association between serum PSA levels and job type. In order to reduce the effect of physical activity to serum PSA levels and evaluate effect of chemical exposure to serum PSA levels indirectly, we subcategorized production workers according to their job and compared serum PSA levels among them. But, there was no significant difference in serum PSA levels between subgroups of production workers (data not shown). According to results of working environment measurement of the factory, the exposed levels of the chemical substances were very low compared to their permissible exposure limit in recent years. Therefore, it is possible that we could not observe elevation of serum PSA levels of production workers and significant differences in serum PSA levels between production workers because exposed levels of the chemical substances might be not enough to elevate serum PSA levels.

In age-stratifying analysis, the likelihood of having an elevated PSA levels and the geometric mean of serum PSA levels of office workers were higher than those of production workers only workers 50 years of age and older. considering that the majority of Korean workers have long years of service in a workplace, there was a possibility of the cumulative effect in accordance with the years of service, However, association between PSA and years of service had not been evaluated directly and this was another limitation of the present study.

Nonetheless, this study is the first research, to the best of our knowledge, to inquire into differences in serum PSA levels between office workers and production workers after adjusting for various factors that may affect serum PSA levels among healthy workers. This study revealed that serum PSA levels were lower in the group of subjects who did regular exercise, and that office workers with low physical activities had higher serum PSA levels as opposed production workers had. In the future, it is necessary to conduct objective and quantitative investigations of detailed job categories, the extent of physical workload and detailed chemical exposure data, and to verify serum PSA levels accordingly. Furthermore, it seems imperative to carry out a future study on whether sedentary workers will be a risk factor in the development of prostate cancer. Owing to the fact that the results of this study exemplified the differences in physical activities between office workers and production workers attributing to each occupation, health promoting inventions such as regular exercise and stretching during work hours would be necessary for office workers.

## Conclusion

The serum PSA level of office workers was higher than that of production workers. This could largely be due to occupational variations in the extent of physical activities between office workers and production workers. The results of this study support the fact that a sedentary job with low physical activities may be the risk factor in the development of a prostate cancer. In this study, the suggestion that job types may affect serum PSA levels, even after adjusting for various confounders, is rather significant in the analysis of serum PSA levels. In the future, it is necessary to conduct an investigation to expose a precise mechanism to bring about differences in serum PSA levels between office workers and production workers. Furthermore, there are studies reporting that higher serum PSA levels, even within the normal range of PSA levels, concur with a high risk of prostate cancer later on. Thus, these data may provide evidence for the importance of health interventions, such as regular exercises and stretching for office workers who have less physical activities on the job.
